# Activation of GSK‐3 disrupts cholinergic homoeostasis in nucleus basalis of Meynert and frontal cortex of rats

**DOI:** 10.1111/jcmm.13262

**Published:** 2017-06-28

**Authors:** Yue Wang, Qing Tian, En‐Jie Liu, Li Zhao, Jie Song, Xin‐An Liu, Qing‐Guo Ren, Xia Jiang, Juan Zeng, Yu‐Tao Yang, Jian‐Zhi Wang

**Affiliations:** ^1^ Department of Pathophysiology School of Basic Medicine and the Collaborative Innovation Center for Brain Science Key Laboratory of Ministry of Education of China for Neurological Disorders Tongji Medical College Huazhong University of Science and Technology Wuhan China; ^2^ Department of Neurology Beijing Chaoyang Hospital Capital Medical University Beijing China; ^3^ Department of Neurobiology Capital Medical University Beijing China; ^4^ Co‐innovation Center of Neuroregeneration Nantong University Nantong JS China

**Keywords:** glycogen synthase kinase‐3, acetylcholine, choline acetyltransferase, nuclear factor‐κB, alzheimer disease

## Abstract

The cholinergic impairment is an early marker in Alzheimer's disease (AD), while the mechanisms are not fully understood. We investigated here the effects of glycogen synthase kinse‐3 (GSK‐3) activation on the cholinergic homoeostasis in nucleus basalis of Meynert (NBM) and frontal cortex, the cholinergic enriched regions. We activated GSK‐3 by lateral ventricular infusion of wortmannin (WT) and GF‐109203X (GFX), the inhibitors of phosphoinositol‐3 kinase (PI3‐K) and protein kinase C (PKC), respectively, and significantly decreased the acetylcholine (ACh) level via inhibiting choline acetyl transferase (ChAT) rather than regulating acetylcholinesterase (AChE). Neuronal axonal transport was disrupted and ChAT accumulation occurred in NBM and frontal cortex accompanied with hyperphosphorylation of tau and neurofilaments. Moreover, ChAT expression decreased in NBM attributing to cleavage of nuclear factor‐κB/p100 into p52 for translocation into nucleus to lower ChAT mRNA level. The cholinergic dysfunction could be mimicked by overexpression of GSK‐3 and rescued by simultaneous administration of LiCl or SB216763, inhibitors of GSK‐3. Our data reveal the molecular mechanism that may underlie the cholinergic impairments in AD patients.

## Introduction

Alzheimer disease (AD) is the most common type of dementia in the elderly. Although the pathogenesis is still elusive, a profound reduction of ACh level and selective loss of cholinergic neurons in specific brain regions have been proposed to be the primary cause for the cognitive impairment in AD patients 1, 2. ACh is synthesized by choline acetyltransferase (ChAT) 3 and degraded by AChE 4. Studies have shown that ChAT is the most specific indicator for monitoring the functional state of cholinergic neurons in nerve system. The activity of ChAT is decreased in the neocortex, which correlates positively with the severity of dementia in AD patients 5. While the AChE may be compensatory decreased in the AD brains 6, the treatments of AD patients so far have been dominated by the application of AChE inhibitors, which offers symptomatic relief by inhibiting ACh turnover and restoring synaptic level of ACh 7. However, not all patients respond equally to the drugs or the cognitive benefit may be of limited duration, and the disease itself does not stop proceeding and irreversibly aggravates at last. Therefore, search for other target is of importance for preserving the cholinergic functions.

Glycogen synthase kinase‐3β (GSK‐3β) has broad physiological and pathological functions, such as regulating glucose metabolism, regulating neurodevelopment as an important component of Wnt/wingless signalling 8, 9 and phosphorylating tau at multiple AD‐associated sites 10, 11. Studies show that neurons undergoing granulovascular degeneration contain active GSK‐3β 12, 13 that is localized in the pretangle neurons, dystrophic neurites and neurofibrillary tangles in the AD brains 14. An increased level of active GSK‐3β coinciding both spatially and temporally with the progression of neurodegeneration has also been observed 15, 16.

As all these studies suggest that GSK‐3 and ChAT are both deeply involved in the AD pathology and symptom, it seems that there is a probable mechanism link of the activated GSK‐3β with impaired cholinergic function in the AD brains. As the direct GSK‐3β activator is still not commercially available, we have developed an indirect GSK‐3β activation model by ventricular infusion of WT and GF‐109203X (GFX), which activates GSK‐3β *via* inhibiting the PI3‐K and PKC, respectively, the kinases contribute to the phosphorylation GSK‐3β at serine‐9 and thus inhibit GSK‐3β 17. Using this rat model, we studied the effects of GSK‐3β on cholinergic functions in the NBM and frontal cortex of the rat brains and the further molecular mechanisms.

## Materials and methods

### Animals and surgical procedures

Male Wistar rats (Grade II, weight 200–250 *g*, 4 months old) were supplied by Experimental Animal Central of Tongji Medical College. All animal experiments were performed according to the ‘Policies on the Use of Animals and Humans in Neuroscience Research’ revised and approved by the Society for Neuroscience in 1995, and the animal study was approved by the Medical Ethics Committee of our college.

Each rat was first anaesthetized by pentobarbital sodium (45 mg/kg, intraperitoneally) and placed on a stereotactic instrument with the incisor bar set 2 mm below the ear bars (*i.e*. flat skull). A 10‐μl syringe (Hamilton) was stereotaxically placed into the left ventricle at the coordinates for bregma and dura of AP (‐0.9), L (1.5) and V (4) and into the left NBM of AP (‐1.4), L (2.5) and V (7) (in mm) after the scalp was incised and retracted. (WT, 100 μM) and GF‐109203X (GFX, 100 μM) or simultaneously LiCl (Li, 200 mM) was injected into the left ventricle (10 μl, all from Sigma‐Aldrich, St. Louis, MO, USA), and same volume of artificial cerebrospinal fluid (aCSF) containing 140 mM NaCl, 3.0 mM KCl, 2.5 mM CaCl_2_, 1.0 mM MgCl_2_, 1.2 mM Na_2_HPO_4_, pH 7.4 was injected as vehicle control 18. The wild HA‐tagged GSK‐3β (GSK‐3β‐HA) plasmid or GSK‐3β adeno‐associated virus (AAV, pAOV‐CMV‐bGlobin‐GSK3β‐eGFP‐3FLAG, NM_001146156) was injected into the NBM (2 μl) of the rats. All surgical procedures were completed under sterile conditions, and penicillin (200,000 U, intramuscularly) was injected to prevent infection.

### Microdialysis and high‐performance liquid chromatography (HPLC) analysis

**Figure 1:**
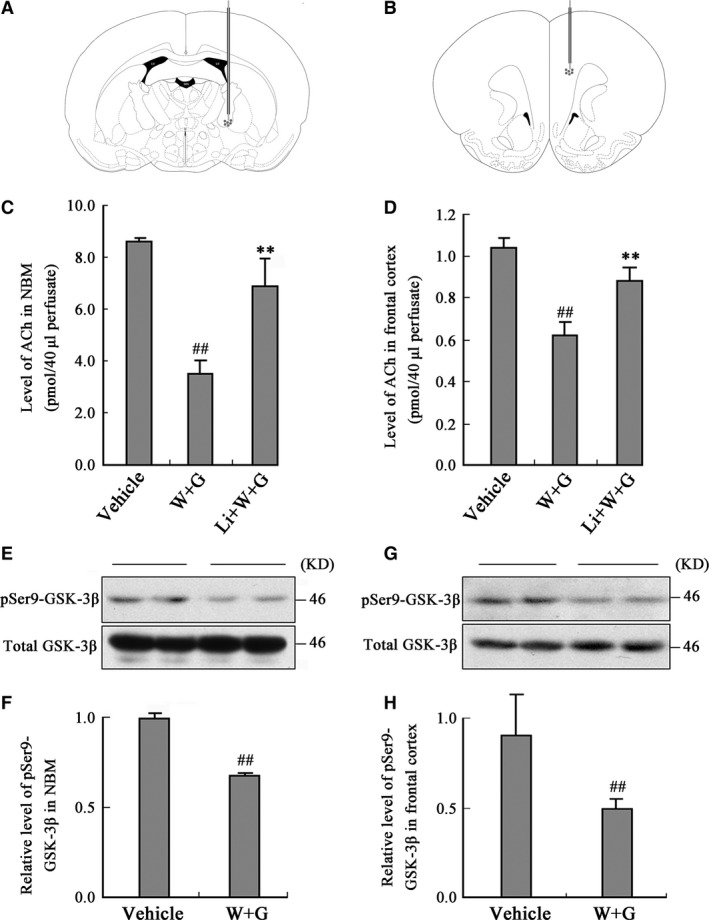
Activation of GSK‐3 decreases ACh level in dialysates of NBM and frontal cortex. The microdialysis probes were implanted into the NBM and frontal cortex of the rats (**A** and **B**) at 30 min. after the left ventricular infusion of WT and GFX (W + G) or simultaneously combined LiCl (Li + W + G) or the vehicle. Then, the ACh level in the dialysates of NBM (**C**) and frontal cortex (**D**) was measured at 24 hrs after ventricular infusion. The Ser9‐phosphorylated GSK‐3β (pSer9‐GSK‐3β) and the total GSK‐3β were measured by Western blotting in NBM (**E**) and cortex (**G**) and relative quantitative analysis (**F**,** H**) at 24 hrs after the infusion. (LV, lateral ventricle; 3V, 3rd ventricle; D3V, dorsal 3rd ventricle; ^##^ *P* < 0.01 *versus* Vehicle; **P < 0.01 *versus* W + G; *n* = 6).

For measurement of ACh in NBM and frontal cortex, microdialysis and HPLC were performed. A microdialysis probe (CMA 11, Carnegie Medicine, Stockholm, Sweden) with 3.0 mm long of dialysis membrane (O.D., 0.24 mm; cut‐off = 20 kDa) was implanted into the left NBM at the coordinates for bregma and dura of AP (‐1.4), L (2.5), V (7) and frontal cortex of AP (2.5), L (1.0), V (3.5) (in mm) according to the stereotaxic atlas of Paxinos and Watson (Fig. 1A and B) 30 min. after stereotaxic infusion. The implanted probes were perfused with Ringer solution (147 mM NaCl, 4.0 mM KCl, 2.4 mM CaCl_2_, pH 7.0) containing 10 μM neostigmine to prevent degradation of ACh, at a perfusion rate of 2 μl/ min. controlled by a micro infusion syringe pump (CMA 102; Carnegie Medicine). The position of the microdialysis probe was verified by histological procedures at the end of each experiment. Recovery rate of ACh of the microdialysis probe *in vitro* is over 96%. The analysis of ACh was carried out at 24 hrs after probe implantation in freely moving animals according to the method described previously 16. In brief, collected samples passed through a reverse‐phase analytical column before entering an immobilized enzyme reactor column, where hydrogen peroxide was produced from ACh. Hydrogen peroxide was detected at a platinum electrode held at a potential of +500 mV relative to an Ag+/AgCl reference electrode. The generated current created a chromatogram peak that was recorded on a flat‐bed recorder and processed by a software program. The chromatogram peak areas are proportional to ACh content in each collected sample. The ACh levels were calculated and then expressed as ρmol/40 ul perfusate for each collected sample.

### ChAT and AChE activity assay

To determine the activities of ChAT and AChE, tissues of NBM and frontal cortex were homogenized in 1% Triton X‐100 at a ratio of 10 ml/g tissue and centrifuged at 4°C 10,000 *g* for 15 min. The supernatants were used for determination of the activities of ChAT and AChE by the radioisotopic method of Fonnum and colorimetric assay, respectively, as previously described 18.

### Nuclear protein extraction and Western blot

NBM extracts were prepared using buffer A 19. Preparation of cytoplasmic and nuclear fractions was performed as previously described 20. The antibodies employed are listed in Table 1. Western blotting was performed as described previously 21. In brief, the NBM, frontal cortex or cell extracts were separated by 10% sodium dodecyl sulphate polyacrylamide gel electrophoresis (SDS‐PAGE) and the protein bands were electrophoretically transferred to polyvinylidene difluoride (PVDF) membranes and immunoblotted with the primary antibodies. Immunoreactive materials were detected using electrochemiluminescence system according to the manufacturer's instruction. The blots were scanned, and the sum optical density was quantitatively analysed by Kodak Digital Science 1D software (Eastman Kodak Company, New Haven, CT, USA).

**Table 1:** Antibodies employed in the study

Antibody	Specific	Type	WB	IHC/IF	Source
PHF‐1	Phosphorylated tau at Ser396/404	mAb		1:250	P. Davies
Ser9‐phospho‐GSK‐3β	Phosphorylated GSK‐3β at Ser9	pAb	1:1000		Cell signalling
GSK‐3β	Total GSK‐3β	mAb	1:1000		Cell signalling
		pAb		1:200	
NF‐κB p100/p52	p100 and p52	pAb	1:1000		Cell signalling
p‐p100	Phosphorylated NF‐κB/p100 at Ser866/870	pAb	1:1000		Cell signalling
HA	Influenza A Hemagglutinin Protein	mAb	1:1000		Cell signalling
ChAT	Choline Acetyl‐transferase	Goat‐ pAb	1:1000	1:200	Millipore
		pAb		1:500	Millipore
pS396	Phosphorylated tau at Ser396	pAb	1:1000	1:500	Biosource
Lamin B_1_	Lamin B_1_ (Clone: L‐5)	mAb	1:1000		ZYMED
SMI33	Non‐phosphorylated neurofilaments	mAb		1:500	Covance
DM1A	α‐Tubulin	mAb	1:1000		Sigma‐Aldrich
β‐actin	β‐actin	mAb	1:1000		Abcam

WB, Western blot; IHC, immunohistochemistry; IF, immunofluorescence; mAb, monoclonal antibody; pAb, rabbit polyclonal antibody; Goat‐pAb, Goat polyclonal antibody.

### Reverse transcription polymerase chain reaction (RT‐PCR)

For RT‐PCR, the rats were decapitated at 24 hrs after the brain infusion and the tissues from the NBM region (50–100 mg) were dissected, lysed by 1 ml Trizol solution (Gibco, Gaithersburg, MD, USA) to extract the total RNA. The total RNA (2 μg) was reverse transcribed in 20 μl of a solution containing 5× reverse transcription buffer, 0.5 μg/μl oligo (dT) 18 primer, 20 u/μl ribonuclease inhibitor, 10 mM dNTP mix and 200 u/μl M‐MuLV reverse transcriptase (Fermentas MBI, Lithuania). Samples were incubated for 5 min. at 70°C, 5 min. at 37°C and then 60 min. at 42°C. The reaction was stopped by heating at 70°C for 10 min. Aliquots of cDNA (5 μl) were amplified with 0.5 U Taq DNA polymerase (Fermentas MBI, Lithuania) in a 20 μl solution containing 10× PCR buffer, 10 mM dNTP mix and 20 pmol of each specific primer. Forward primer of ChAT (GenBank XM_224626): 5′‐TTT GCT CGG CAG CAC TTC‐3′ (643–660); reverse primer of ChAT: 5′‐TCG TTG GAC GCC ATT TTG‐3′ (996–1013). Forward primer of β‐actin (GenBank NM_031144): 5′‐CAT CAC TAT CGG CAA TGA GC‐3′ (822–841); Reverse primer of β‐actin: 5′‐GAC AGC ACT GTG TTG GCA TA‐3′ (961–980). Amplification reactions were overlaid with light mineral oil and held at 94°C for ‘hot‐start’ PCR for 5 min. and then run in an automated thermal cycle for 35 cycles. Each cycle consists of 94°C for 1 min., 55°C for 1 min. and 72°C for 1 min., with a final extension for 3 min. at 72°C. The electrophoretically isolated PCR products were photographed with a Kodak Digital Science Scanner.

### Cell studies

The human embryonic kidney 293 (HEK293) cells were cultured in DMEM supplemented with 10% foetal bovine serum at 37°C in 5% CO_2_. The cells were transiently transfected with the GSK‐3β‐HA plasmid using lipofectamine 2000 transfection kit according to the manufacturer's instructions.

Primary neuronal cultures were prepared from embryonic 17 of Sprague–Dawley rat as described previously with some modifications 22. Briefly, basal forebrain was dissected from the whole‐brain tissues and digested in 0.25% trypsin (Invitrogen, Grand Island, NY, U.S.A.) at 37°C for 15 min. Dissociated neurons were plated into poly‐D‐lysine (Sigma‐Aldrich) coated coverslips in 12‐well plate. After 7 days, we added 1 ul GSK‐3β AAV or vector virus in relative wells and cultured for an additional 7 days. After fixing by 4% paraformaldehyde for 30 min., we carried out the immunofluorescence staining.

For fluorescence recovery after photobleaching (FRAP) experiments, wild Neuro‐2a (N2a) cells were seeded in 4 cm dishes in DMEM medium with 10% foetal bovine serum (all from Gibco) and transfected with composite rat ChAT‐GFP‐expressing plasmid (pEGFP‐C2‐RChAT) when cells coved 60% area. Thirty‐six hours after transfection, we treated cell with DMSO or 1 μM WT and GFX or simultaneously combined 5 μM SB216763 (all from Sigma‐Aldrich) for 1 hr, then replaced medium by DMEM (without carbonate and phenol red), and supplemented with 20 mM HEPES. Imaging was performed with a Zeiss equipped with a preheated 37°C chamber using the 488 nm Ar laser. Ten prebleach images were acquired with 1% laser intensity, followed by bleaching with 100% laser intensity. Bleaching was targeted to regions in long branching. After bleaching, 150 images were taken with a scanning speed of 60 Hz with 1% laser intensity. FRAP recovery rate was determined after full‐scale normalization. Rate constant, recovery half time and mobile fraction of pEGFP‐C2‐RChAT were calculated by the software for FRAP. The fluorescence recovery intensity was normalized to the prebleach fluorescence intensity.

### The rat brain studies of morphology

At 24, 48, 72 and 96 hrs following the ventricular infusion of WT and GFX, the rat brains were fixed *in situ* for 20 min. by transcardial perfusion with Zamboni's solution. Then, the brains were isolated and fixed in the same solution for another 12 hrs at 4°C and sliced coronally about 20 μm thickness with a freezing microtome (CM1900, Leica Microsystems, Wetzlar, Germany).

For immunohistochemistry, the sections containing NBM and frontal cortex were incubated for 48 hrs at 4°C with primary antibodies PHF‐1 (1:250), pS396 (1:500) and ChAT (1:500). The slices were developed with HistostainTM‐SP kits (ZEMED, South San Francisco, CA, USA) and visualized with 3,3′‐Diaminobenzidine (DAB) staining.

For double‐labelling immunofluorescence, the brain sections containing NBM collected under identical conditions. After blocking with 3% BSA for 1 hr, the sections were then incubated for 48 hrs at 4°C with a mixture of anti‐non‐phosphorylated neurofilament (SMI33) and anti‐ChAT or anti‐phospho‐tau at Ser396 epitope (pS396) antibodies. Then, the immunoreactivity was probed with relevant conjugated secondary antibodies. Fluorescent‐stained sections were examined using an Olympus FV500 confocal laser scanning microscope (Olympus, Tokyo, Japan).

The Bielschowsky's silver staining was carried out as described 23. Briefly, the sections were placed in 3% silver nitrate solution (37°C) for 30 min. in dark and reduced for 5 min. with 10% formaldehyde. Then, the sections were treated with ammonium silver alcohol solution for 5 min. and soaked in 8% formaldehyde until the sections become dark brown.

The Nissl staining was carried according to the standard procedure 16. To further assess any possible atrophy and neuronal loss, magnetic resonance imaging (MRI) and magnetic resonance spectroscopy (MRS) were applied 16. For transmission electron microscopy (TEM), the ultrathin sections stained with uranyl acetate and lead citrate were observed by TEM.

### Statistical analysis

Data were expressed as x ± SD and analysed using spss 20.0 statistical software (spss Inc., Chicago, IL, USA). The one‐way ANOVA procedure followed by LSD's post hoc tests was used to determine the different means among groups (*P* < 0.05).

## Results

### Activation of GSK‐3 decreases ACh level in NBM and frontal cortex measured by microdialysis and HPLC

To explore the role of GSK‐3 in cholinergic function, we implanted the microdialysis probes into different brain regions and measured ACh level in the dialysate after ventricular infusion of WT and GFX or simultaneously combined LiCl, an inhibitor of GSK‐3. We found that the ACh level in the dialysates decreased prominently at 24 hrs after ventricular infusion of WT and GFX, simultaneous infusion of lithium attenuated the reduction of ACh level in NBM (Fig. 1C) and the frontal cortex (Fig. 1D).

To validate the increase of GSK‐3 activity, we measured the relative levels of Ser9‐phosphorylated GSK‐3β (pSer9‐GSK‐3β, inactive form) and the total GSK‐3β. We found that the level of pSer9‐GSK‐3β decreased with unchanged total GSK‐3β level in NBM (Fig. 1E) and the frontal cortex (Fig. 1G) after infusion of WT and GFX by quantitative analysis (Fig. 1F and H).

### Activation of GSK‐3 inhibits ChAT but not AChE in NBM and frontal cortex

**Figure 2:**
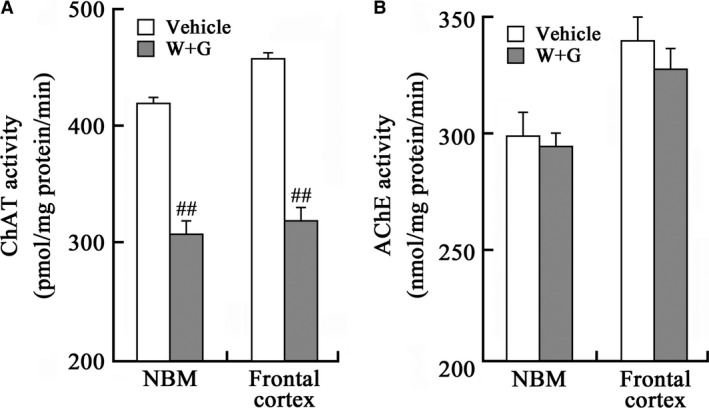
Activation of GSK‐3 inhibits ChAT without affecting AChE. The rats were infused through left ventricle with WT and GFX (W + G) or the vehicle for 24 hrs; then, the activity of ChAT (**A**) and AChE (**B**) in NBM and the frontal cortex extracts was measured as shown. (##, *P* < 0.01 *versus* Vehicle; *n* = 6).

Choline acetyltransferase and AChE are, respectively, responsible for the biosynthesis and degradation of ACh. Therefore, we measured the activity of ChAT and AChE after infusion of WT and GFX for 24 hrs. The activity of ChAT in both NBM and frontal cortex was decreased prominently (Fig. 2A), whereas the activity of AChE was not altered (Fig. 2B). These data suggest that the inhibition of ChAT is involved in the decreased ACh level induced by GSK‐3 activation instead of AChE.

### Activation of GSK‐3 causes axonal accumulation of ChAT in cortex neurons

**Figure 3:**
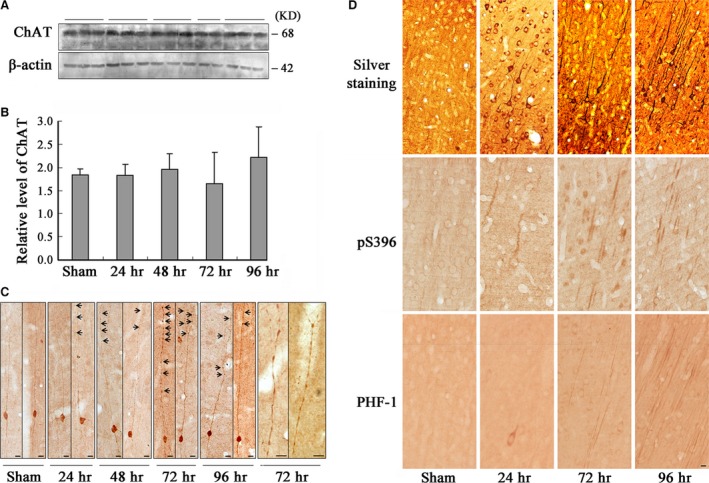
Activation of GSK‐3 causes axonal accumulation without affecting the level of ChAT in frontal cortex neurons. The rats were infused through left ventricle with WT and GFX; then, the frontal cortex slices and extracts were prepared at different time‐points as indicated (24–96 hrs). The level of ChAT was measured by Western blotting (**A**) and quantitative analyses normalized against β‐actin (**B**). Distribution of ChAT in the frontal cortex layer III pyramidal neurons was observed by immunohistochemistry, and the amplified images showing ChAT accumulation in axonal swellings at 72 hrs after infusion (**C**, arrowheads indicate axonal swellings). The enhanced argyrophil substances in the cell body and proximal neurites of the frontal cortex neurons at 24 hrs and the tangle‐like structures were shown at 72 and 96 hrs after infusion by Bielschowsky's silver staining (**D**, Silver staining). Similar pattern of the phosphorylated tau at Ser396 and PHF‐1 epitopes was shown by immunohistochemistry (**D**, pS396 and PHF‐1). (Bar = 10 μm; *n* = 6).

To explore the underlying mechanisms involved in the decreased ChAT activity, we first detected the expression level of ChAT in the frontal cortex. Unexpectedly, we did not observe significant decrease of ChAT level by Western blotting with internal control of β‐actin (Fig. 3A and B). Then, we detected the cellular distribution of ChAT by immunohistochemistry staining. We observed that ChAT was uniformly distributed in both cell bodies and axons in rats of sham group (Fig. 3C, sham); however, an enhanced ChAT staining was detected in the distant axonal swellings (Fig. 3C, black arrow). The accumulation of ChAT was especially prominent at 72 and 96 hrs after the infusion of WT and GFX (Fig. 3C, 72 and 96 hrs).

To validate the axonal pathology, we used Bielschowsky's silver staining. Enhanced argyrophil substances were shown in the cell bodies and proximal neurites at 24 hrs after infusion of WT and GFX, and the argyrophil substances migrated to the apical neurites and massive tangle‐like structures were detected in the cortical neurons at 72 and 96 hrs after WT and GFX infusion (Fig. 3D, silver staining). Similar pattern of distribution was found when we detected with antibodies of phosphorylated tau by immunohistochemistry (Fig. 3D, pS396 and PHF‐1). The distribution of both argyrophil tangle‐like structures and phosphorylated tau coincided with the progression of axonal swellings, especially at 72 hrs, which indicated that ChAT transport impairment might be due to these tauopathies. Fluent axonal transportation of ChAT is closely associated with its normal physiological function on ACh synthesis 3; hence, these data together contributed to the mechanism of decreased ACh level observed in the frontal cortex.

### Activation of GSK‐3 inhibits expression and causes accumulation of ChAT in NBM neurons without cell death

**Figure 4:**
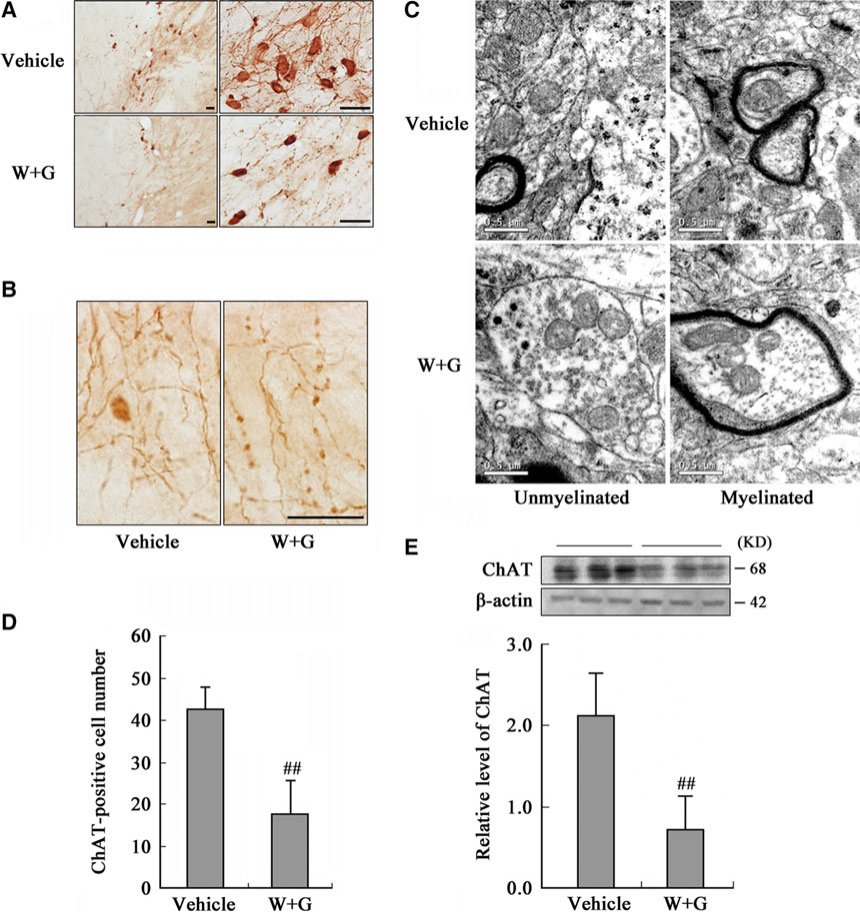
Activation of GSK‐3 inhibits expression and causes accumulation of ChAT in NBM. The rats were infused through left ventricle with WT and GFX (W + G) or the vehicle for 24 hrs. (**A**) The distribution of ChAT in NBM neurons (Bar = 20 μm). (**B**) The amplified images showing axonal swellings (Bar = 20 μm). (**C**) Axon swelling measured by TEM (Bar = 0.5 μm). (**D**) The quantitation of ChAT‐positive neurons by stereological analysis. (**E**) The protein level of ChAT was measured by Western blotting, with β‐actin as an internal control. (^##^ *P* < 0.01 *versus* Vehicle; *n* = 6).

To explore the underlying mechanisms involved in the decreased ChAT activity in NBM, we detected the expression and distribution of ChAT. By immunohistochemical staining, a remarkably decreased immunoreactivity of ChAT at the neuronal processes (Fig. 4A) and number of ChAT‐positive cells were detected at 24 hrs after infusion of WT and GFX (Fig. 4A and D). Additionally, a prominently decreased ChAT level was detected by Western blotting (Fig. 4E). By immunohistochemistry, we also found enhanced ChAT staining in the cell bodies and axonal swellings in NBM (Fig. 4A and B). To explore the possible contents in the axonal swellings, we used TEM. Significant axonal swellings, including abnormal accumulation of mitochondria, vesicles, organelles and dense bodies, were observed in both unmyelinated and myelinated neurons (Fig. 4C).

**Figure 5:**
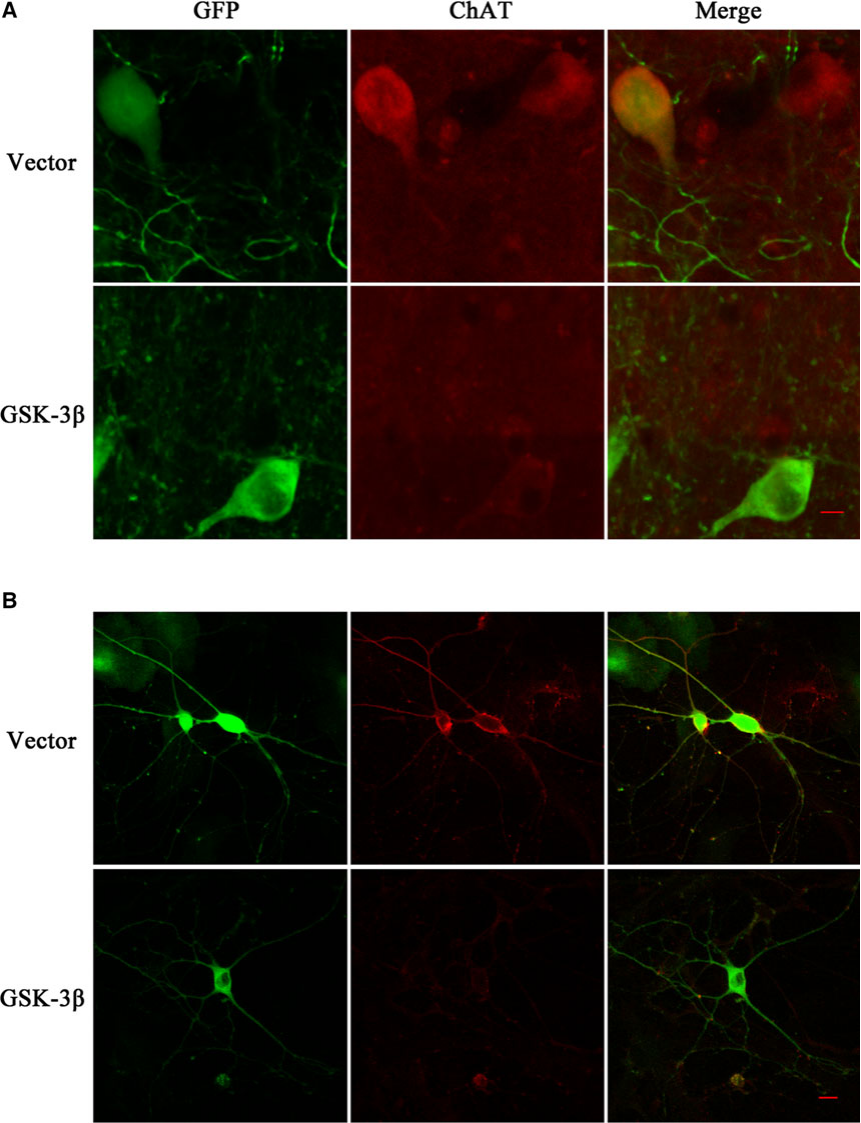
Overexpression of GSK‐3 inhibits ChAT protein level in NBM and primary cultured neurons of rat. We infected GSK‐3β AAV or vector virus in the rat NBM region for a month *in vivo* (**A**, green) and primary cultured neurons of the forebrain for 7 days *in vitro* (**B**, green), respectively. GSK‐3β with green fluorescence was detected by confocal laser scanning microscope. The red fluorescence represented ChAT protein in neurons. The pictures on the right column were the merged results of green and red fluorescence in relative groups. (Bar = 10 μm).

Moreover, we infected NBM neurons *in vivo* (Fig. 5A) and primary neurons *in vitro* (Fig. 5B) with GSK‐3β AAV, and also found reduced ChAT level. These results confirmed that both activation and overexpression of GSK‐3 down‐regulated ChAT expression level in cholinergic neurons.

**Figure 6:**
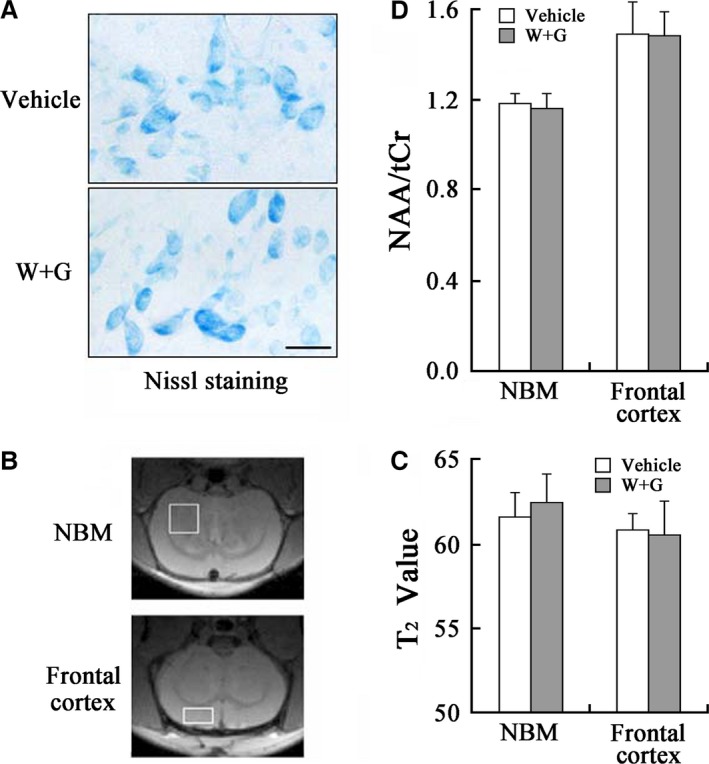
Temporal alterations of the cell numbers and viability induced by WT and GFX in NBM and frontal cortex. (**A**) Nissl staining in NBM at 24 hrs after ventricular infusion. (**B**) The representative image of the rat brain demonstrated by MRI with placement and locations of voxel from NBM (3 mm × 3 mm × 1.5 mm) and frontal cortex (3.0 mm × 1.5 mm × 3.0 mm) respectively. (**C** and **D**) Quantitative analysis of T_2_ value and ratio of N‐acetyl‐aspartate to total creatine (NAA/tCr) in NBM and frontal cortex. The central of regional of interesting (ROI) in quantitative T_2_ map was same in all images, and the thickness was 0.8 mm. The spectra were shown with similar linewidth and with the amplitude adjusted using tCr peak at 3.03 ppm (Repetition time = 1.5 sec., Echo time = 136 ms, Number of averages = 512).

As the number of ChAT‐positive neurons was significantly decreased in NBM (Fig. 4A and D), we also examined whether this decline was caused by cell death. We first did Nissl staining and found that the total cell number in NBM was not altered by stereological analysis at 24 hrs after ventricular infusion of WT and GFX (Fig. 6A). We also used MRI and MRS to detect any possible atrophy or cell loss (Fig. 6B–D). The T_2_‐weighted images in the focal area inside the brain were unchanged, and no visible ventricle augmentation or atrophy in NBM was detected by MRI (Fig. 6B). Similarly, no obvious alteration in T_2_ value and the ratio of NAA/tCr were observed after WT and GFX infusion measured by MRS (Fig. 6C and D), and consistent result was found in frontal cortex (Fig. 6B–D). These data indicate that the loss of ChAT‐positive neurons is consequent with the decreasing ChAT expression rather than a general cell death.

**Figure 7:**
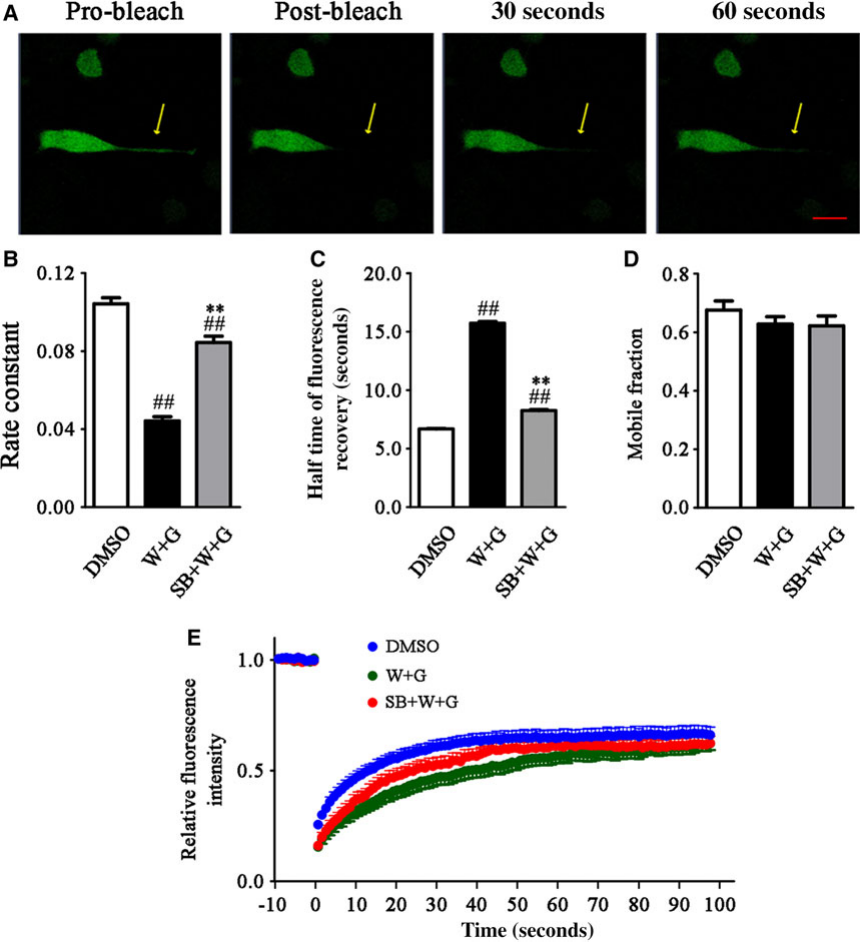
WT and GFX reduced the mobility of transfected ChAT in live wild N2a cells. Wild N2a cells were transfected with pEGFP‐C2‐RChAT plasmid for 36 hrs and then treated with 1 μM DMSO or 1 μM WT and GFX (W + G) or simultaneously combined 5 μM SB216763 (SB + W + G) for 1 hr. Fluorescence images of FRAP processes of expressive ChAT were recorded using a confocal microscope. Bleaching was targeted to regions in long branching. The pro‐bleaching and post‐bleaching images were showed before the drug treatment, which indicated successful expression and normal migration of transfected ChAT in the live cell (**A**). The rate constant (**B**), half time of fluorescence recovery (**C**) and mobile fraction (**D**) of ChAT were calculated by the software for FRAP. The fluorescence recovery of ChAT after photobleaching was also recorded (**E**). The fluorescence recovery intensity was normalized to the pro‐bleach fluorescence intensity. (^##^ *P* < 0.01 *versus* DMSO; **P < 0.01 *versus* W + G; Bar = 20 μm).

To verify the axonal transport impairment, we used FRAP to detect whether WT and GFX treatment affected the transport process of the expressive ChAT in the transfected N2a cells with pEGFP‐C2‐RChAT plasmid. For FRAP experiments described in method, we could observe the fluorescence in marked area disappeared (bleach) and slowly recovered (post‐bleach) in N2a cells’ long processes (Fig. 7A). The rate constant of the WT and GFX group was significantly lower than that of the control group (Fig. 7B), and the half recovery time was significantly longer than that of the control group for 10 sec. (Fig. 7C). These data indicate that the transport rate of ChAT in N2a cells is decreased after treatment of WT and GFX. In order to further prove the inhibition of ChAT transport is caused by GSK‐3 activation, we gave WT and GFX treatment with the GSK‐3 inhibitor SB216763 to N2a cells simultaneously. The results showed that the rate constant increased significantly after SB216763 treatment, but did not return to the control group level (Fig. 7B). The fluorescence recovery time decreased to about 8 sec., but still slightly higher than the control group (Fig. 7C). The mobile fraction dynamics of the three groups was not significantly different (Fig. 7D). The record of the fluorescence change with time curve also showed that the fluorescence intensity of WT and GFX infusion group (Fig. 7E, W + G) represented the slowest recovery, while the recovery rate of simultaneously SB216763 infusion group (Fig. 7E, SB + W + G) was obviously faster than it but still slower than the control group (Fig. 7E, DMSO), which indicating that SB216763 could partly reverse the inhibitory effect of GSK‐3 on the transport of ChAT. These results confirmed that GSK‐3 activation can specifically inhibit the ChAT transport in the N2a cell processes.

### Activation of GSK‐3 decreases mRNA level of ChAT through the phosphorylation and cleavage of nuclear factor‐κB in NBM

**Figure 8:**
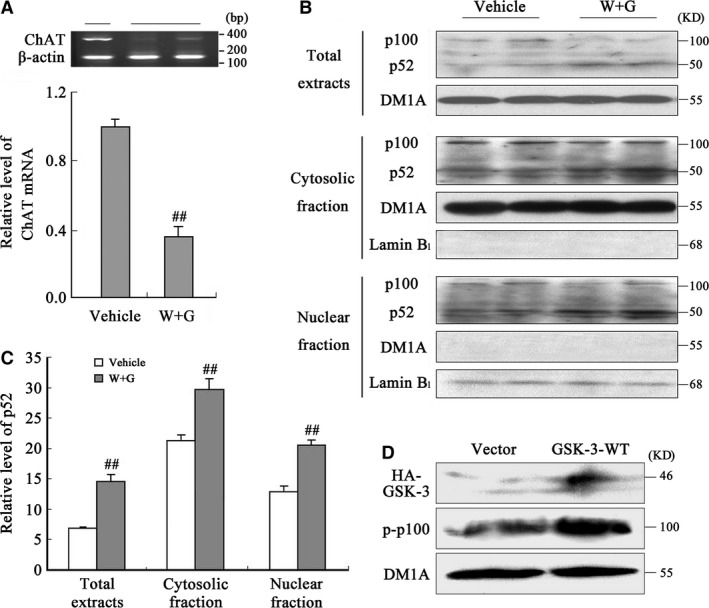
Activation of GSK‐3 stimulates phosphorylation and cleavage of NF‐κB/p100 into p52 and consequently decreases the mRNA level of ChAT in NBM. (**A**) The mRNA level of ChAT in NBM was measured by RT‐PCR at 24 hrs after infusion of WT and GFX (W + G) or the vehicle. Quantitative analysis of band intensity was normalized against β‐actin. The total, cytosolic and nuclear fractions extracted from NBM were used for Western blotting (**B**) and quantitative analysis (**C**) of p100 and p52. DM1A and lamin B_1_ were used, respectively, as internal controls of cytosolic and nuclear proteins. (**D**) HEK293 cells were transfected with wild GSK‐3β‐HA plasmid for 24 hrs; then, the level of phosphorylated p100 (p‐p100) was measured by Western blotting. (^##^ *P* < 0.01 *versus* Vehicle; *n* = 6).

As ChAT level decreased in NBM neurons, we found the mechanism by series of experiments as follows. The tissues from NBM region were dissected at 24 hrs after ventricular injection, and the extracts of total RNA were prepared for RT‐PCR test. We found that the mRNA of ChAT obviously decreased after WT and GFX infusion compared with Vehicle treatment, with β‐actin as internal control (Fig. 8A). To explore the underlying mechanisms involved in the decreased ChAT mRNA level induced by GSK‐3 activation, we measured the cleavage of nuclear factor‐κB (NF‐κB)/p100, a factor can inhibit synthesis of ChAT in the nucleus through its degradation product NF‐κB/p52 24. We observed that the level of p52 increased prominently accompanying with a decreased p100 in the total NBM extracts with WT and GFX exposure for 24 hrs (Fig. 8B, total extracts). Similar results were found in both cytosolic (Fig. 8B) and nuclear (Fig. 8B) fractions. Relative quantitative analysis was also shown (Fig. 8C). The results suggest an increased cleavage of p100 into p52, which consequently facilitates the translocation of p52 into nuclei and inhibits ChAT synthesis.

Phosphorylation of p100 at Ser866/870 triggers the cleavage itself 25. To explore the involvement of p100 phosphorylation in its cleavage in present study, wild GSK‐3β‐HA was expressed in HEK293 cells and the phosphorylation level of p100 was measured. We found that the phosphorylated p100 at Ser866/870 significantly increased in the cells transfected with GSK‐3β‐HA plasmid (Fig. 8D), suggesting that p100 phosphorylation was concerned with the increased GSK‐3β activity, which may underlie the increased cleavage of p100.

### Increased GSK‐3 activity causes ChAT accumulation in cell body through modulating phosphorylation level of tau and neurofilaments in NBM

**Figure 9:**
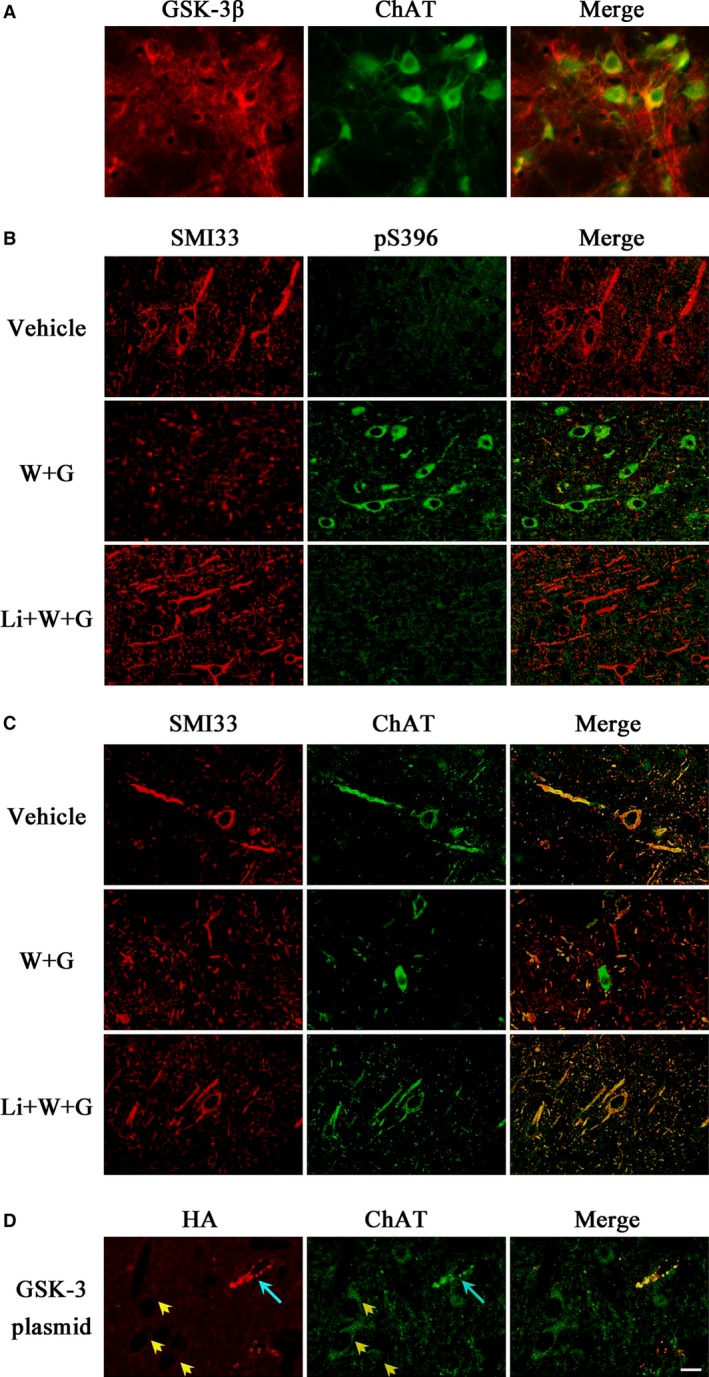
WT and GFX causes ChAT accumulation accompanied with the phosphorylation disorder of tau and neurofilaments in NBM. The simultaneous expression of GSK‐3β and ChAT was observed in NBM neurons of normal rat (**A**). Vehicle or WT and GFX (W + G) or simultaneously combined LiCl (Li + W + G) in a total volume of 2 μl was injected directly into NBM for 24 hrs, respectively. The expression and cellular distribution of phosphorylated tau (pS396), non‐phosphorylated neurofilaments (SMI33) and ChAT were measured by immunofluorescent staining (**B** and **C**). Wild GSK‐3β‐HA plasmid mixed with lipofectamine 2000 was injected into the NBM for 24 hrs, then the accumulation of ChAT was successfully detected in transfected cells (**D**, blue arrow), but not in the untransfected cells (yellow arrowhead). (Bar = 20 μm; *n* = 6).

The simultaneous cytoplasmic expression of GSK‐3β and ChAT was observed in NBM neurons of normal rat (Fig. 9A), which gave support to the modulation effect of GSK‐3β to ChAT. To explore the possible mechanism of ChAT transport retardation in NBM, we injected directly WT and GFX or simultaneously combined LiCl or GSK‐3β‐HA plasmid into the NBM, the region with enriched cholinergic neurons. By immunofluorescent staining, we found increased phosphorylated tau (Fig. 9B, pS396) and decreased non‐phosphorylated neurofilaments (Fig. 9B and C, SMI33) at 24 hrs after WT and GFX infusion. The ChAT distributed evenly in the neurons and well merged with the non‐phosphorylated neurofilaments in vehicle group, while the cytoplasmic accumulation of ChAT was detected in WT and GFX group (Fig. 9C, ChAT). It suggests that ChAT distribution is related to the phosphorylation level of tau and neurofilaments.

Simultaneous application of lithium partially improved the abnormal cellular distributions of ChAT and phosphorylation level of tau and neurofilaments (Fig. 9B and C, Li + W + G).

By direct injection of GSK‐3β‐HA plasmid into the NBM, we observed that the neurons expressing HA‐tagged GSK‐3β showed accumulation of ChAT (Fig. 9D, blue arrow), but the accumulation was not observed in the neurons without expression of the GSK‐3β in the same visual field (Fig. 9D, yellow arrowhead), which demonstrated the GSK‐3β‐dependent ChAT misdistribution.

## Discussion

Impairment of cholinergic neurotransmission is a well‐established fact in AD patients, but the upstream factor leading the impairment is still not understood. Using a microdialysis system, we found in the present study that activation of GSK‐3 by ventricular infusion of WT and GFX led to a remarkable reduction of ACh in the dialysates of NBM and frontal cortex in rats. As ChAT and AChE are, respectively, the rate‐limiting enzymes for the synthesis and degradation of ACh, we detected the activity of the two enzymes and only the decreased ChAT was observed, suggesting that GSK‐3 reduces ACh level through interrupting the synthesis but not the degradation system. Further studies demonstrated that activation of GSK‐3 caused severe axonal accumulation of ChAT in both frontal cortex and NBM regions due to abnormal phosphorylation of tau and neurofilaments and suppressed the expression of ChAT in NBM neurons. Our data provide direct evidence showing that activation of GSK‐3 reduces ACh level through interrupting the axonal transport or/and expression of ChAT.

Increasing evidences imply that GSK‐3 may be involved in cholinergic function. The expression of GSK‐3β is restricted in the neurite structures of the basal forebrain cholinergic neurons in young rats, whereas it is confined to the cell bodies in the aged rats 26. The degeneration of forebrain cholinergic neurons induced by cholinergic immunotoxin 192‐IgG saporin is mediated by an increased GSK‐3β activity and the GSK‐3β inhibitor LiCl can partly protected cholinergic neurons 27. GSK‐3β can phosphorylate and inactivate pyruvate dehydrogenase (PDH), which regulates ACh synthesis by converting pyruvate into acetyl‐CoA in mitochondria. In cholinergic neurons, Aβ impairs ACh synthesis through inactivating PDH by GSK‐3β 28. In PC12 cells, inhibition of PI3‐K blocks the nerve growth factor‐induced elevation of ACh level that may involve GSK‐3 activation 29. Furthermore, activation of GSK‐3 and suppression of cholinergic function have been observed in the AD brains 10, 30. Based on these reports and our current observations, we propose that GSK‐3 activation may be upstream for the damnified ACh synthesis system. As is well‐known, some other neurotransmitters were involved in the AD pathogenesis including dopamine, 5‐hydroxytryptaminem, glutamate, noradrenaline, γ‐aminobutyric acid and so on. However, the relationship between these neurotransmitters and GSK‐3 and definite mechanism are not elucidated. To our knowledge, only dopamine 31 and 5‐hydroxytryptamine 32 showed primary regulatory links, which deserved more research.

**Figure 10:**
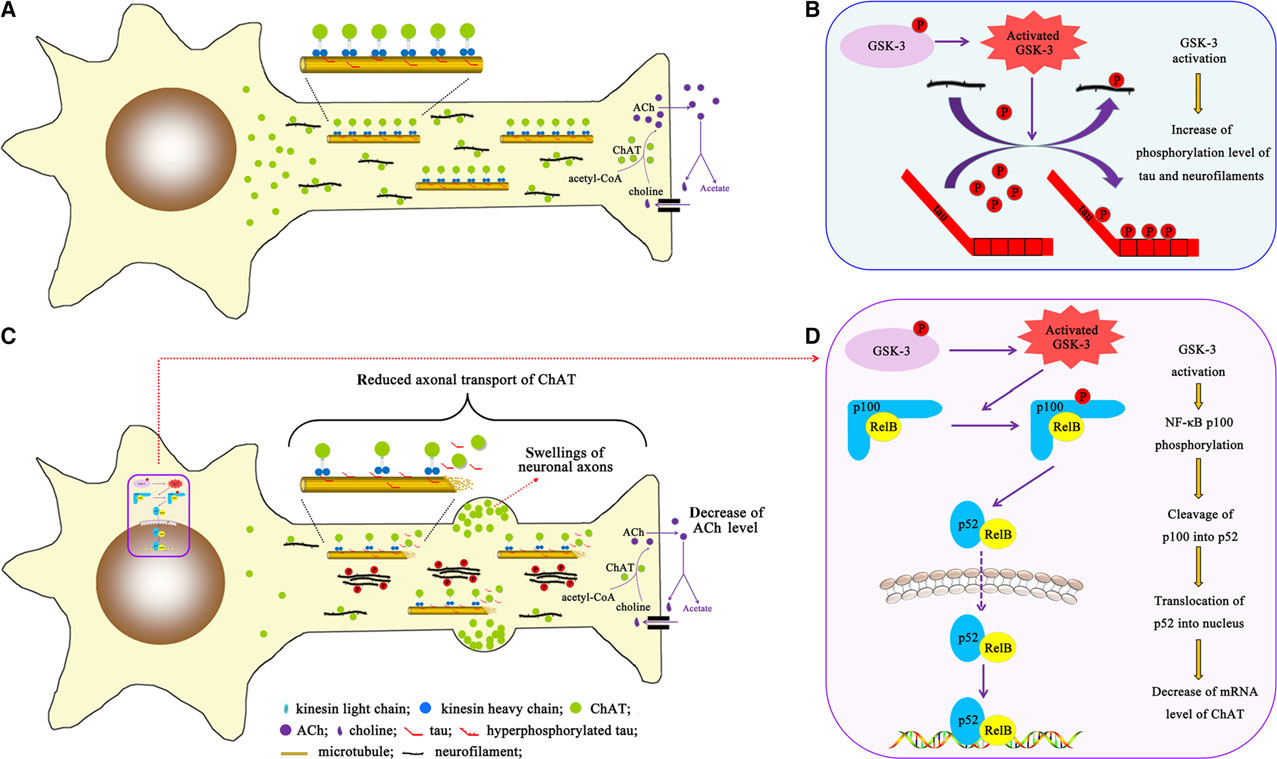
Schematic diagram of the underlying mechanism of cholinergic function modulation by GSK‐3 activation. (**A**) ChAT is synthesized in the cell body of cholinergic neurons and transported to the synaptic terminals along the microtubules and neurofilaments for catalyzing the biosynthesis of ACh. GSK‐3 was activated *via* dephosphorylation at Ser9 epitope or overexpression in our present study; then, the microtubules and neurofilaments were hyperphosphorylated (**B**), which resulted in microtubule stability decline and neurofilaments aggregation (**C**). Both morphological abnormity prevented the smooth ChAT transport and led to the swelling formation in the axons, which correspondingly decreased the ChAT concentration in the neurite terminals and down‐regulated the ACh level ultimately (**C**). Activated GSK‐3 phosphorylated NF‐κB/p100 and promoted its cleavage into p52. The active p52 migrated into the nucleus and inhibited mRNA level of ChAT (**C** and **D**). Our experiments directly illustrated the underlying mechanism about regulatory relationship between GSK‐3 and ChAT.

Choline acetyltransferase plays a crucial role in maintaining the ACh level in the nerve terminals. In primary neuronal cultures, the alteration in ACh synthesis parallels change of ChAT activity independent of high‐affinity choline transport 33. It is well recognized that a normal axonal transport is the precondition to ensure ChAT transport from the cell body to the axon terminal, and thus to catalyze ACh synthesis at the terminals 3. In present study, we observed severe accumulation of ChAT in the axonal swellings in both frontal cortex and NBM neurons upon activation of GSK‐3, suggesting impairment in axonal transport of ChAT (Fig. 10).

The neurofilament proteins are normally synthesized in cell bodies and transported into and through axons by axonal transport 34. There is evidence that this transport of neurofilaments is impaired in numerous neurodegenerative diseases due to the aberrant phosphorylation, aggregates and subsequent accumulation 35. GSK‐3 has been shown responsible for the neurofilaments phosphorylation resulting in impaired axonal transportation 36. Additionally, our previous study has found that non‐phosphorylated neurofilaments are prerequisite for unimpeded ChAT axonal transportation in rat striatum. The GSK‐3 activation decreases the level of non‐phosphorylated ‘individual’ neurofilaments and leads them to form more ‘bundle’ ones, which will be more difficult to be transported to the neuronal distal end 18. Similarly, we found non‐phosphorylated neurofilaments‐dependent well‐balanced distribution and transport of ChAT in NBM in present study, which further confirms the involvement of neurofilaments in mechanism of ChAT transport deficit (Fig. 10).

Furthermore, GSK‐3 also phosphorylates tau, which is well‐known for disrupting microtubule assembly and axonal transport. Elevated tau phosphorylation decreases its microtubule binding and bundling and increases the number of motile tau particles, which affects the axonal transport 37. The tau phosphorylation mediated by GSK3β is associated with proper anterograde transport 38. Therefore, we speculate that hyperphosphorylation of tau induced by GSK‐3 activation may be involved in the impaired axonal transport of ChAT (Fig. 10). In present study, we indeed found tau hyperphosphorylation in neurons of NBM, which is well related to the ChAT transport deficit. Additionally, it is also noticed that the ChAT swellings became more severe in the distal end of the frontal cortical neuronal axons that were in parallel with the appearance of numerous tangle‐like argyrophil structures at 72 and 96 hrs after ventricular infusion WT and GFX; however, there was no obvious cell loss although the pathology was apparent. This observation illustrated that the tangle‐like structures by formation of the hyperphosphorylated tau destroyed the regular ChAT transportation without cell death, which is in consistent with that seen in Huntington disease 39.

GSK‐3 affects the expression level of several synaptic structural proteins, including synapsin I, PSD93, NMDA receptor 2A/B 40. In the present study, we also observed that activation of GSK‐3 suppressed the expression of ChAT in the NBM, source of cholinergic systems from the basal forebrain. Then, we further explore how GSK‐3 activation affects the expression of ChAT. It has been reported that the cleavage of NF‐κB from p100 to p52 and the nuclear translocation of p52 negatively regulate ChAT expression 24, and the cells with GSK‐3β deficits fail to activate an NF‐κB‐dependent reporter gene efficiently 41. To explore the involvement of NF‐κB processing in the GSK‐3‐induced inhibition of ChAT expression, we measured NF‐κB subunit level. The significantly decreased level of p100 and increased level of p52 as well as nuclear translocation were observed after ventricular infusion of WT and GFX. These results suggest that GSK‐3 may stimulate p100 processing and p52 nuclear translocation resulting in inhibition of ChAT expression (Fig. 10). A previous study demonstrates that subtype of NF‐κB can be phosphorylated by many kinases, including GSK‐3β 42. Moreover, NF‐κB/p100 may be phosphorylated at Ser866/870 leading to trigger the cleavage itself 25. To confirm the role of GSK‐3 in phosphorylating p100 and explore the mechanism of p100 cleavage, we transfected GSK‐3β plasmid into the HEK293 cells and found prominently increased level of the phosphorylated p100 at Ser866/870 epitopes.

Taken together, we have found in the present study that activation of GSK‐3 reduces ACh level in both NBM and frontal cortex of rat with the mechanisms involving disruption of axonal transport and suppression of ChAT expression (Fig. 10).

## Conflict of interest

The authors declare no conflicts of interest.
